# The Interplay Between Prostate Cancer Genomics, Metabolism, and the Epigenome: Perspectives and Future Prospects

**DOI:** 10.3389/fonc.2021.704353

**Published:** 2021-09-29

**Authors:** Reema Singh, Ian G. Mills

**Affiliations:** ^1^ Nuffield Department of Surgical Sciences John Radcliffe Hospital, University of Oxford, Oxford, United Kingdom; ^2^ Patrick G Johnston Centre for Cancer Research, Queen’s University of Belfast, Belfast, United Kingdom; ^3^ Centre for Cancer Biomarkers, University of Bergen, Bergen, Norway; ^4^ Department of Clinical Science, University of Bergen, Bergen, Norway

**Keywords:** prostate cancer, metabolism, epigenetics, mitochondria, TCA cycle

## Abstract

Prostate cancer is a high-incidence cancer, often detected late in life. The prostate gland is an accessory gland that secretes citrate; an impaired citrate secretion reflects imbalances in the activity of enzymes in the TCA Cycle in mitochondria. Profiling studies on prostate tumours have identified significant metabolite, proteomic, and transcriptional modulations with an increased mitochondrial metabolic activity associated with localised prostate cancer. Here, we focus on the androgen receptor, c-Myc, phosphatase and tensin Homolog deleted on chromosome 10 (PTEN), and p53 as amongst the best-characterised genomic drivers of prostate cancer implicated in metabolic dysregulation and prostate cancer progression. We outline their impact on metabolic function before discussing how this may affect metabolite pools and in turn chromatin structure and the epigenome. We reflect on some recent literature indicating that mitochondrial mutations and OGlcNAcylation may also contribute to this crosstalk. Finally, we discuss the technological challenges of assessing crosstalk given the significant differences in the spatial sensitivity and throughput of genomic and metabolomic profiling approaches.

## Introduction

Prostate cancer (PCa) is the most common cancer affecting men in the developed world. The incidence is the second highest after lung cancer in men worldwide (1, 2).

Deciphering the functional impact of prostate cancer genomics on disease progression has been a challenge in comparison to other cancer types for many reasons including sample accessibility and the limited availability of model systems. Multi-focal sampling of prostate cancer in patient samples with downstream DNA and RNA sequencing have revealed both inter- and intra-patient heterogeneity in primary tumours and metastatic samples (3, 4). Despite this heterogeneity, it has been possible to sub-type prostate cancers based not only on gene fusion status but also on the abundance of mutations associated with biological drivers of the disease, and in particular mutations affecting androgen receptor (AR) signalling), PI 3-Kinase/Akt, and DNA repair pathways (3).

## Genomic Features of Prostate Cancer

In considering the crosstalk between genetic changes in prostate cancer and metabolic dysregulation, it is helpful to focus on some of the principal oncogenic drivers—AR activity, c-Myc amplification and overexpression and mutations in phosphatase and tensin homolog deleted on chromosome 10 (PTEN) and in TP53.

## AR

Over the decades, a focus on targeting the androgen receptor (AR) signalling axis to block AR *via* androgen deprivation therapy and AR antagonists has been a conventional therapy in PCa. Aberration in AR ranges from point mutations such as W741R, V757A, R846G, H874Y, and T877A in ligand binding region of the AR imparting insensitivity towards AR antagonists (1). In addition, deletions in the region of G589-A628 has been identified in patients with CRPC, disrupting second zinc-finger domain of AR, developing resistance to AR antagonists or AR targeted therapies (1). By using ChIP-seq and transcriptomic profiling networks of AR, target genes have been identified in cell lines and in tumour samples (2). These datasets have provided insight into AR crosstalks with other transcription factors and regulated biological processes. Recent transcriptomic and cistromic studies have revealed AR as a modulator of autophagy and DNA repair (3–7). Massie et al. employed a combination of transcript profiling and ChIP-seq to identify androgen receptor target genes and pathways in prostate cancer cell lines (8). Through a meta-analysis of clinical transcriptomic data and subsequent validation using immunohistochemistry, the authors identified calcium/calmodulin-dependent kinase kinase 2 (CAMKK2) as a clinically relevant regulator of metabolism. They went on to knockdown CAMKK2 and also inhibit this kinase with a small molecule inhibitor, which impaired tumorigenesis in a prostate cancer xenograft model. Pairing these interventions with ^13^C-glucose metabolic flux analysis using mass spectroscopy, they showed that targeting CAMKK2 inhibited the incorporation of hydrocarbons into TCA cycle metabolites and amino acids. This work illustrates the use of genomics and metabolomics to identify metabolic regulators that are affected by AR activity. Other studies have shown that AR-associated gene targets include key components/enzymes of glucose homeostasis, mitochondrial respiration, and fatty acid oxidation (9–14).

Pathway enrichment analysis on AR-regulated gene networks has unearthed enrichments for metabolic processes amongst which the most prominent are lipid synthesis and degradation pathways (15). Importantly, the vast majority of AR-regulated metabolic enzymes are cytosolic or associated with organelles other than mitochondria; however, high rates of metabolic activity arising from AR-regulated pathways feed metabolites into mitochondria. Important examples of lipid-metabolising enzymes that are AR-dependent include FASN, ELOVL5, and ACACA (acetyl-CoA carboxylase alpha) (16). The precise functional effects of aberrant lipid metabolism remain to be determined but include changes in membrane fluidity and the generation of acetyl CoA to support the post-translational modification of proteins (acetylation and glycosylation), prominent amongst which are histones that are of relevance to the crosstalk between metabolism and the epigenome (see below) (17). In addition, a number of other important oncogenic drivers of prostate cancer also sustain aberrant lipid metabolism (see below); as such, this biology is arguably a convergence point for prostate cancer tumorigenesis and, consequently, may offer opportunities for the development of new treatments and repurposing of existing drugs (8, 18).

## c-Myc

Myc is copy number amplified and overexpressed in poor-prognosis prostate cancer and exerts an impact on tumour metabolism. It has been shown to affect expression levels of enzymes of oxidative/glycolytic pathway including hexokinase 2, phosphofructokinase, enolase 1, and lactate dehydrogenase A and also GLUT1 levels (19–21). Interestingly, many of these effects are synergistic with the hypoxia-inducible factor 1 (HIF1) function (22). c-Myc also regulates glutamine transporter and mitochondrial glutaminase GLS1 expression through miRNA23a/b and subsequently enhances glutamine metabolism (23, 24). c-Myc may play a significant role in global metabolic reprogramming, such as fuelling citric acid cycle intermediates into anabolic pathways on similar line with AR (25). In addition, c-Myc expression is inversely correlated to AR activity, emphasizing the precise balance and regulation of oncogenic transcription factors thresholds playing a significant role in PCa cells (26). Interestingly, an integrative analysis of metabolomics based on mass spectroscopy revealed differential expression of metabolites; association of AKT1 and MYC activation correlated with accumulation of metabolites of aerobic glycolysis and dysregulated lipid metabolism in human tumours, mouse models, and also in cultured cells (RWPE-1 cells), establishing the oncogene-associated metabolic signatures in PCa (27). In a recent *in vivo* study, a high-fat diet (HFD) led to both metabolic dysregulation and upregulated the MYC transcriptional cascade. These changes favoured H4K20 histone hypomethylation at the promoter regions of MYC-regulated genes, supporting enhanced cell proliferation and tumour growth. This study exemplifies the link between the activity of oncogenic transcription factors and feedback effects on the epigenetic landscape of cancer genomes, a theme we explore further in this review (28).

## PTEN

PTEN is a well-established tumour suppressor exhibiting both protein and lipid phosphatase activities. Loss of PTEN function is common in various cancers including bladder, brain, and prostate cancers, often through the deletion of a single gene copy of PTEN at chromosomal location 10q23 (29, 30). It is a negative regulator of oncogenic PI3K/AKT signalling network and plays a vital role in both lipid and glucose metabolism including mitochondrial functions (31, 32). *In vivo* studies with transgenic models overexpressing PTEN showed an overall change in the metabolic profile with increase in mitochondrial oxidative phosphorylation and coupled with reduction in glucose and glutamine uptake (33).

PTEN has been used as the basis for the transgenic modelling of prostate cancers, and this has revealed that deletion of this tumour suppressor leads to the activation of SREBP1, a transcription factor that regulates lipogenic genes (34). This transcriptional program is enhanced by co-deletion of PTEN with other factors (for example PML1), and tumorigenesis in these models, analogous to c-Myc, is enhanced through a high-fat diet. In a separate study using a prostate-specific conditional PTEN-null (PTEN−/−) transgenic mouse model of cancer, increased pyruvate dehydrogenase activity was shown to be required for tumorigenesis. Genetic ablation of pyruvate dehydrogenase A1 (Pdha1) in PTEN −/− tumors inhibited tumour growth, and this was associated with the reduced expression of lipogenic genes, which were components of a gene network regulated by sterol regulatory element-binding transcription factor (SREBF). Importantly, nuclear Pdha1 was found to sustain this transcriptional activity by supporting histone H3K9 acetylation at sites bound by SREBF1, putatively supporting its transcriptional activity. Interestingly, whereas the knockdown of Pdha1 in PTEN −/− prostate cancer cells reduced acetylation of histone H3 Lys9 (H3K9ac) at these sites, it had not impact at E2F1 binding sites associated with cell cycle progression genes (35). This specificity, and in fact the molecular basis of the crosstalk between metabolite pools and site-specific, as opposed to global, changes in chromatin modifications or DNA methylation remain largely undefined in this and other published studies identifying similar interplays. One example is the nuclear contribution of ATP-citrate lyase to the provision of acetyl-CoA for histone acetylation in lung cancer (36), and others will be highlighted in the course of this article. Overall, these examples of crosstalk suggest that metabolic reprogramming may sustain and be sustained by transcription factors reinforced by metabolically dependent chromatin modifications.

## p53

p53 mutations are amongst the most common features of various cancer types including treatment-resistant prostate cancer (37–39). It is often marked by a loss of one allele and inactivity of the second allele resulting in p53 inactivity resulting in cell cycle deregulation and genomic stability (40, 41). Gain-of-function mutations in p53 can also confer oncogenic properties and resistance towards therapeutics (42). p53 regulates metabolism by inhibiting the expression of genes of pentose phosphate shunt pathway and counteracting Myc- and HIF-induced glycolytic flux (43). p53 can also impair nuclear factor kappa B-dependent glucose uptake and glycolysis by repressing the expression of glucose transporters, GLUT1/4 and GLUT3 (44).

p53 also regulates glutamine metabolism through activation of phosphate-activated mitochondrial glutaminase (GLS2) and mitochondrial glutaminase promoting ATP generation *via* oxidative phosphorylation (45). In addition, p53 inhibits AR activity; also, a loss of p53 function enhances Myc activity in PCa (46, 47). This may be partly explained by the enhanced amino acid metabolism and mitochondrial activity arising from p53 deletion. For example, p53 deletion results in mitochondrial biogenesis and in mitochondrial dysfunction mediated by PGC-1α mitochondrial in PC3 prostate cancer cells (48). p53 loss also results in enhanced serine/glycine biosynthesis and changes in one-carbon metabolism that support DNA methylation and nucleotide production (49).

Many of the transcriptional effects of p53 on metabolic gene expression are likely to arise from changes in an impact on the activity of chromatin regulators and hence histone methylation and acetylation. p53 gain of mutants modulates chromatin regulatory genes, including the methyltransferases mixed-lineage leukaemia family of histone methyltransferases 1 and 2 (MLL1 and MLL2) and acetyltransferase monocytic leukaemia zinc finger protein (MOZ) resulting in genome-wide upregulation of histone methylation and acetylation (50). p53 negatively modulates H2Bub1 expression independently of the role of p53 as a transcription factor, establishing it as a significant epigenetic modulator (51).

**Figure 1:**
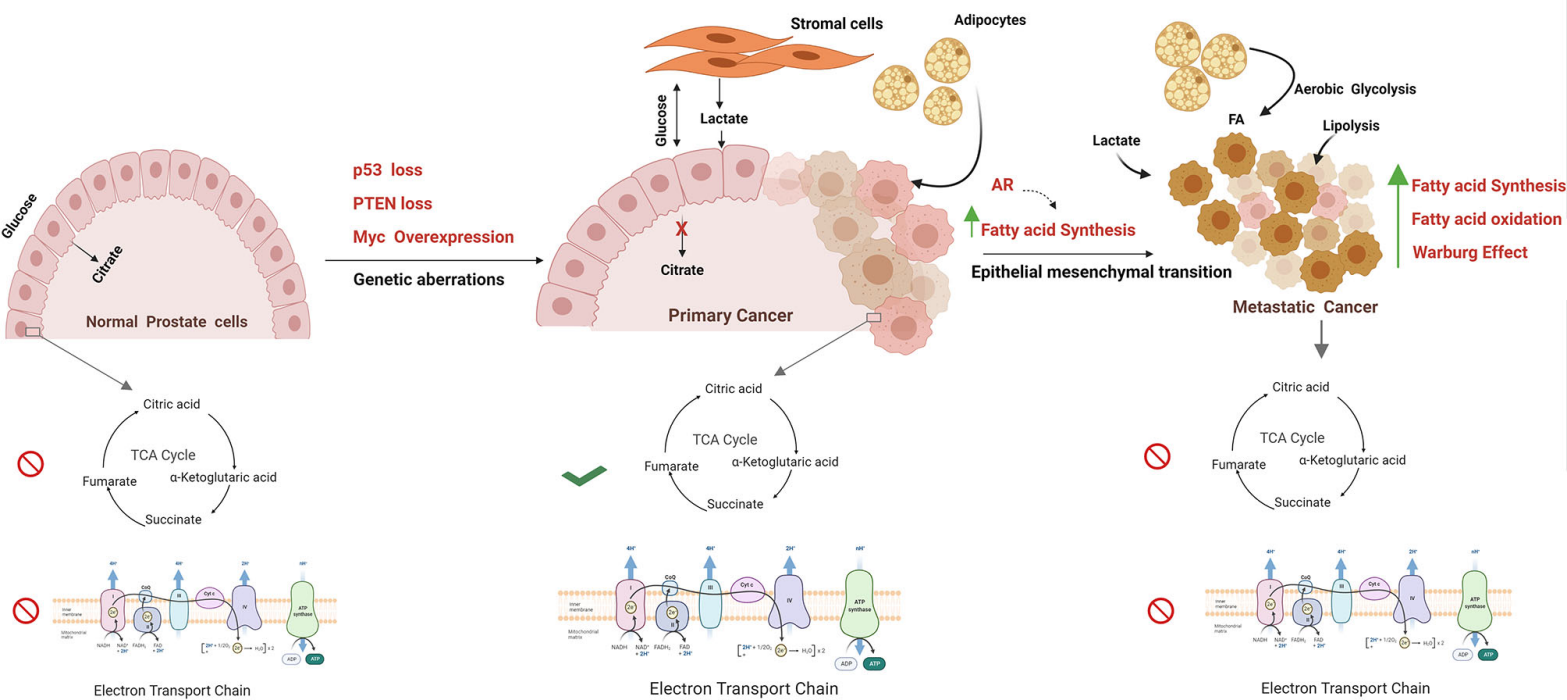
Metabolism alterations in prostate cancer cell. The figure represents the metabolic alterations during prostate cancer metastasis. The normal prostate epithelial cells are characterised with increased release of citrate in seminal fluid, abbreviated Krebs cycle, and reduced oxidative phosphorylation rate. Normal prostate cells undergo transformation due to genetic aberrations; for example, MYC overexpression, PTEN loss, p53 loss, mutations, and other tumor suppressors result in activation of TCA cycle and oxidize citrate and generate Acetyl CoA for lipid biosynthesis. Besides, these fatty acids are fuelled into TCA cycle through lipolysis of adipocytes. Increased Warburg effect is a common feature in metastatic PCa with high lactate secretion, provided by cancer-associated fibroblasts. Progression towards mCRPC is marked by epithelial to mesenchymal transition. In metastatic stage, energy demand is met by both fatty acid oxidation and fatty acid synthesis. The figure has been drawn using Biorender software.

As highlighted, these oncogenic drivers have a significant impact on the balance between glycolytic and TCA cycle activity in cancer cells. It is worth reflecting on the fact that in normal prostate cells, both the TCA cycle and OXPHOS are impeded, and there is a net secretion of citrate. By contrast, in PCa, OXPHOS activity is increased in cancer cells in localised disease and a new dynamic exists between cancer cells and the tumour microenvironment. This dynamic entails increased production and turnover of citrate, a reduction in citrate secretion, and lactate exchange between tumour and stromal cells (52). Lactate exchange sustains both catabolism and anabolism and supports OXPHOS activity in cancer cells. As prostate cancer progresses to a treatment-resistant, metastatic state, TCA cycle activity is once again impaired, and cancers develop a Warburg-like metabolism otherwise termed aerobic glycolysis. So far, no single study has evaluated these metabolic states alongside the genomic landscape of tumour cells and other cell types within the tissue (**Figure 1**).

## The Crosstalk Between Epigenomes and Metabolism

**Figure 2:**
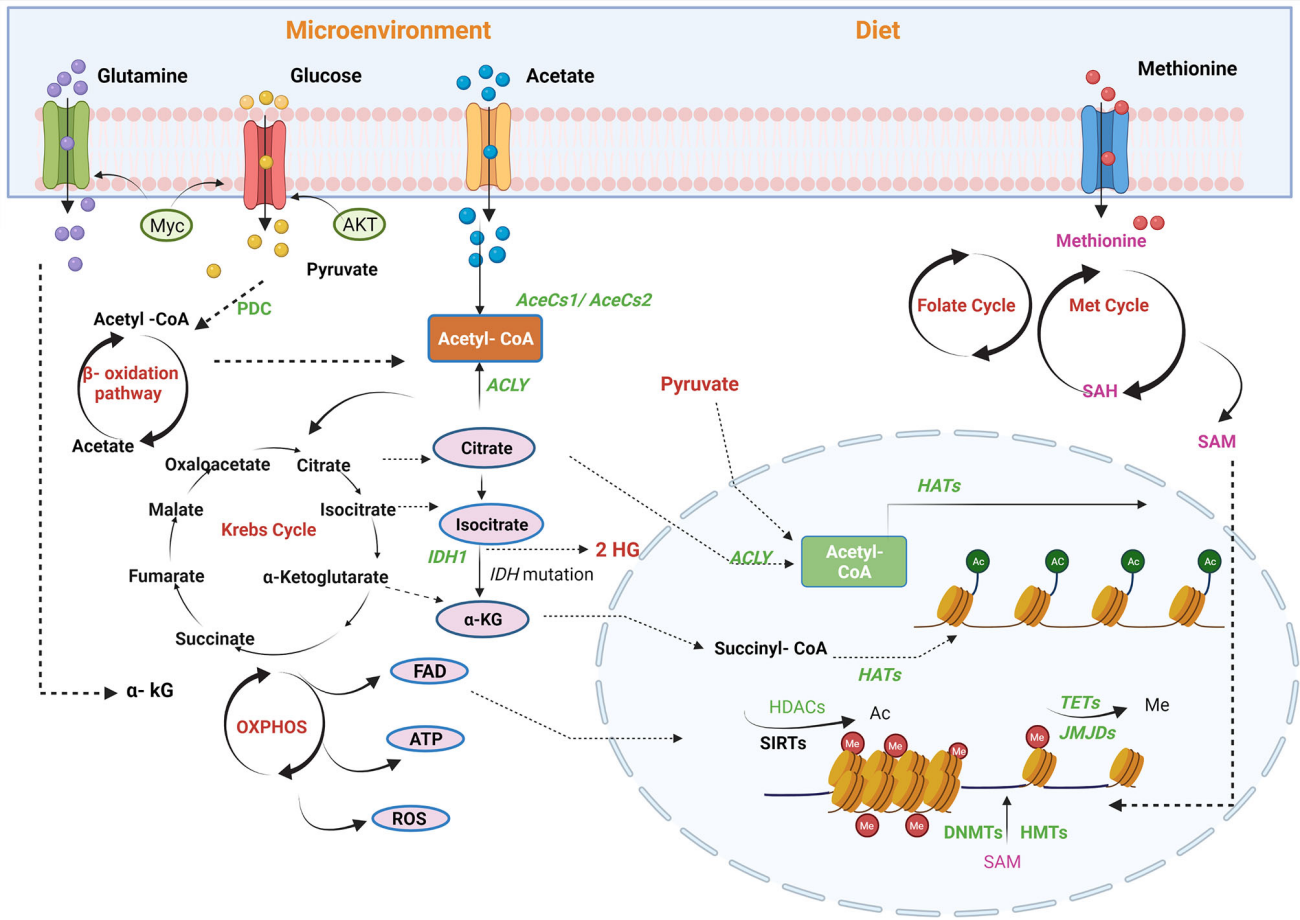
Regulation of PCa epigenome by metabolic pathways. Metabolites are the mediators of epigenetic regulation through mitochondrial and nucleus crosstalk. Different factors contribute to epigenetic regulation, for example, microenvironment and availability of nutrients through diet. Acetyl-CoA, a connecting link between different metabolic pathways, provides acetyl groups in the cell, catalysed by histone acetyltransferases (HATs) for histone modifications. In mitochondria, acetyl-CoA is generated *via* fatty acid oxidation, catalysis of pyruvate-by-pyruvate dehydrogenases complexes (PDC), and by utilising acetate by acyl-CoA synthetase short-chain family member 1/2 (AceSS1/AceSS1). Furthermore, TCA cycle metabolites, pyruvate, citrate, and acetate translocates into nucleus to generate acetyl-Co A pool by pyruvate dehydrogenase complex, ACLY and AceSS2, respectively. Another TCA cycle metabolite, α-ketoglutarate (KG), also translocates into the nucleus and is utilised by histone demethylases [Jumonji C domain-containing (JMJD)] and DNA demethylase [ten–eleven translocation (TET)]. In addition, isocitrate also diffuses into the cytosol and is converted to α-KG by isocitrate dehydrogenase enzyme. FADH2, a by-product of β-oxidation, is oxidised by electron transport chain into flavin adenine dinucleotide (FAD), which translocates to the cytosol or nucleus. The amino acid cycles, namely, methionine and folate, generate an S-adenosyl methionine (SAM), a methyl group donor utilised by DNA methyltransferase (DNMT) and histone methyltransferases (HMTs). Acetyl-CoA, acetyl-coenzyme; ACLY, ATP-citrate lyase; SIRTs, sirtuins; TETs, ten–eleven translocation family; α-KG, alpha-ketoglutarate; HDAC, histone lysine deacetylase; DNMT, DNA methyltransferase; ATP, adenosine triphosphate; FAD, flavin adenine dinucleotide; TCA, tricarboxylic acid; DNMT, DNA methyltransferase; JMJD, Jumonji C domain-containing histone demethylase. The figure has been created with Biorender.com.

We have previously outlined the contribution of a number of transcription factors, including p53, c-Myc, and hypoxia-inducing factor (HIF), to prostate cancer progression acting in part by regulating the expression of metabolic enzymes. Metabolism can, in turn, alter the accessibility of chromatin to these factors supplying or restricting hydrocarbon adducts required for epigenetic alterations, principally consisting of histone modifications and DNA methylation (53) (**Figure 2**). This dynamic relationship may allow cells to rapidly adjust their transcriptional programs in response to treatment or environmental stress and provide the basis for plasticity and the emergence of new cell lineage characteristics in resistant cells (54).

## DNA and Histone Methylation

DNA methylation involves the addition of a methyl group to the 5′-carbon of cytosine in CpG dinucleotide sequences catalysed by a family of DNA methyltransferases (DNMTs). CpG islands are CpG-rich regions, located proximal to promoter region of genes with high expression. The DNA methylation/demethylation can result in inhibition/activation of transcription of genes, and in prostate cancer, malignant transformation is a common feature as a result of DNA methylation (55). A study in a castration-resistant prostate metastasis exhibited a novel epigenomic subtype associated with hypermethylation and somatic mutations in TET2, DNMT3B, IDH1, and BRAF and also identified differential methylation associated with transcriptional expression of AR, ERG, and Myc oncogenic drivers (56).

In assessing early-stage drivers of prostate cancer based on their incidence, epigenetic alterations, and particularly DNA methylation change, and gene fusions are far more prevalent than somatic point mutations or indeed copy number alterations in most genomic loci (57). For example, GSTP1 promoter hypermethylation is a feature of >60% of localised prostate cancers, and by contrast, TP53 point mutations/copy number deletions are associated with approximately 10% of localised prostate cancers (58–60). Epigenetic changes are also known to be affected by perturbations in metabolic pools and, in the case of methylation, by changes in TCA cycle metabolites and metabolites associated with serine, glycine, and polyamine biosynthesis and the one-carbon cycle (61). In the TCGA prostate cancer dataset, the prostate tumours with the highest genome-wide levels of DNA hypermethylation carry IDH1 point mutations, and this suggests that perturbations in alpha-ketoglutarate and succinate levels associated with these mutations disrupting methylation status by inhibiting TET enzyme activity and the conversion of methyl- to 5-hydroxymethylcytosine marks (62). Ten–eleven translocation (TET) proteins are dioxygenases involved in the regulation of demethylation by oxidizing 5-methylcytosine to 5-hydroxymethylcytosine. Both expression and activity of TET proteins are deregulated in various ranges of cancers including prostate cancer. Mutations in TET2 and reduced TET have been associated with poor prognosis in in prostate cancer (63). The activity of the TET enzyme is regulated by metabolites from TCA cycle and oxygen pool. Dioxygenases utilise a metabolite, 2-oxoglutarate (2-OG), as an essential cofactor that is generated by isocitrate dehydrogenases (IDH).

Isocitrate dehydrogenase, an NADP^+^-dependent enzyme, which decarboxylates isocitrate to α-ketoglutarate in the TCA cycle, has been found to carry heterozygous mutations in the prostate including other cancers such as acute myeloid leukaemia (AML) (64). Another study by Ghiam et al., using mutational and array comparative genomic hybridization analyses, has identified *IDH1* mutations (R132, R172, or R140 mutations) in localized prostate cancer (PCa) (65). IDH mutations can impair dioxygenase activity by restricting the availability of this cofactor and in turn enhancing the steady-state levels of DNA methylation genome-wide. This DNA hypermethylation phenotype, relative to the cohort as whole, has been observed in the small percentage of prostate cancer TCGA cases carrying IDH mutations (66, 67). A second impact of IDH mutations on DNA methylation has been deciphered through the use of pre-clinical models, which show that these mutations lead to the accumulation of an oncometabolite, R (−)-2-hydroxyglutarate (2HG) (68, 69). *In vitro*, ectopic expression of IDH1 mutants generate high levels of an oncometabolite, (R)-2HG, which perturbs DNA and histone methylation by inhibiting α-ketoglutarate-dependent enzymes including TET dioxygenases and histone demethylases Jumonji 2 (JMJD2) and JMJ C domain-containing histone demethylase-1 (JHDM1) (70–72). In addition, the perturbation in TCA cycle metabolites arising from these mutations also leads to the stabilization of HIF-1α under normoxic conditions and enhanced glycolytic activity (73).

DNA methyltransferase 1 (DNMT1), the methyltransferase enzyme that modulates gene expression by methylating cytosine residues within CpG dinucleotides, regulates DNA methylation and is found to be overexpressed in higher in localized, metastatic, and hormone-resistant PCa compared with benign prostate hyperplasia (BPH) (74–77). In PCa, high DNMT1 expression has been associated with high grade/stage cancers (78).

Overall, although methylation changes are high-incidence events in localised prostate cancer, yet there is limited evidence to suggest that genome-wide increases in methylation are prognostics. By contrast, chromatin relaxation and increased enhancer activity, associated with histone acetylation, are a feature of castrate-resistant prostate cancer (79).

## Histone Acetylation

Histone acetylation occurs on lysine residues and reflects the balance of activity of histone acetyltransferases (HATs) and histone deacetylases (HDACs) (80, 81). HATs utilise acetyl-CoA derived from a number of metabolic processes, and consequently, nutrient availability and utilisation can, in principle, affect the steady-state levels of histone acetylation in tumours (82–84). For example, in PTEN-null prostate cancers, nuclear pyruvate dehydrogenase A1 (PDHA1) is a source of acetyl-CoA for histone H3 K27 acetylation and, as consequence, sustains SREBP1 transcriptional activity (85). Pyruvate dehydrogenase complex (PDH) is another example of an enzyme that links glycolysis and the TCA cycle. It converts pyruvate, a glycolytic metabolite to acetyl CoA in the mitochondria (86). The pyruvate dehydrogenase complex (PDC), however, has also been found in the nucleus in prostate cancer (87). Mitochondrial PDH regulates the availability of citrate in mitochondria for lipid biosynthesis, whereas nuclear regulates expression of sterol regulatory element-binding transcription factor (SREBF)-target genes by mediating histone acetylation. In addition, an amplified expression of PDHA1, both at protein and gene level, have been reported in prostate tumours (87). *PDHA1 gene* knockout in prostate cancer cells developed alterations in tumor cell metabolism with an increase in expression of glutaminase1 (GLS1) and glutamate dehydrogenase1 (GLUD1), leading to an increase in glutamine-dependent cell survival (88). All these outcomes indicate that PDH supports prostate tumorigenesis not only by regulating lipid biosynthesis but also by utilising alternate metabolic pathways for cell survival.

Altered acetyl-CoA levels significantly affect the substrate specificity of CBP and p300 acetyltransferases. For example, at a low concentration of acetyl CoA, p300 has the highest specificity for histone H4K16, for which specificity is 10^18^-fold higher than CBP (89). The acetyl-CoA-producing enzyme ATP-citrate lyase (ACLY) regulates histone acetylation levels in a nutrient-dependent manner in cells (36, 89). The location of ACLY in both nucleus and cytosol further suggests that it plays a role in both histone acetylation and lipid biosynthesis (90). In a limited nutrient environment (low glucose levels), cancer cells can still modulate and increase acetyl CoA pool by AKT (S473)-mediated ACLY phosphorylation and upregulates histone acetylation marks in prostate tumors (91).

Whilst a number of recent pre-clinical molecular studies have highlighted important crosstalk between acetyl-CoA production and histone acetylation, contributing tumorigenesis, nothing similar has yet been possible in the study of clinical disease. This in part is due to the dynamic changes that occur in the metabolic states of tumours, which makes it technically very challenging to generate robust high-throughput metabolomic data on a similar scale and resolution to genomic data (refer to *Future Perspectives* for more discussion). Given the significant progress that has been made in developing epigenetic drugs as cancer therapeutics, it is of course vital to learn more about this interplay because greater functional and clinical understanding could support ultimately the use of metabolic drugs as sensitising agents. Prostate cancers are also susceptible to inhibitors of fatty acid oxidation, a prominent source of acetyl CoA, and examples include etomoxir and perhexiline (92). These are examples of co-dependencies between metabolic and epigenetic activities that maybe amenable to combinatorial treatments if patient stratification is possible. If we consider mitochondrial activity as a primary determinant of the availability of acetyl-CoA within cancer cells, then what is the evidence that mitochondrial mutations exist within tumour cells and also have an impact on the epigenome?

## Crosstalk Between Mitochondrial Activity and the Epigenome

Unlike many cancer types, OXPHOS activity is enhanced in the transition from benign/untransformed tissue to cancer in the prostate gland—as discussed above. Given the prominent role that mitochondria play in the turnover of acetyl-CoA, this change might be expected to correlate with increased acetylation. Thus far, no translational studies have attempted to assess the relationship between mitochondrial activity and histone acetylation or indeed chromatin relaxation. A number of studies have, however, shown that mitochondrial mutations accumulate during prostate cancer progression.

The human mitochondrial DNA encodes 13 polypeptides crucial for oxidative phosphorylation, 22 transfer RNA molecules, and 2 ribosomal RNA molecules essential for mitochondrial translational machinery, and the rest is encoded by nuclear genome (93–95). This coordinated expression of subunits of mitochondrial proteins and replication machinery through mitochondrial and nuclear genes is regulated by a bidirectional flow of intermediates (metabolites) and polypeptides including enzymes and is the key of co-regulated biologies of nuclear and mitochondrial processes (96). In addition to somatic mutations in nuclear genome, the mitochondrial genome shows a 55-fold higher incidence of mutation rate in comparison to nuclear genome in PCa (97). A sequencing study identified mutational hotspots in the mitochondrial genomes of 384 prostate cancer and went on to associated mitochondrial mutational burden with Myc amplification and disease recurrence in a subgroup of poor-prognosis patients (98). The functional basis for this relationship remains undefined; however, preclinically researchers have been able to deplete prostate cancer cells of mitochondrial DNA using sub-toxic doses of DNA-damaging agents, creating so-called rho-null derivatives. These depleted cell lines have reduced levels of histone acetylation (principally histone H3K9, H3K18, and H3K27), which suggests that mitochondrial content could affect the epigenetic landscape of tumours (99). In other studies, focussing on the impact of mitochondrial mutations on the tumorigenic potential of prostate cancer cells, it has been possible to use rho-null derivatives as acceptor lines in cell fusion experiments to re-complement the cells with mutated mitochondrial genomes. Since the acceptor and wild-type lines remain isogenic in their autosomal genomes, the enhanced metastatic potential of the resultant cybrids has been attributed to the mutations present in the mitochondrial genome. Using this principle, Petros et al. generated cybrids in PC3 cells with mitochondrial DNA (mtDNA) ATP6 T8993G mutations and engrafted them into immune-compromised mice, resulting in enhanced tumorigenesis compared to wild-type cells and increased production of reactive oxygene species (100). Whilst these distinct studies highlight the impact of mitochondrial activity on the epigenome and of mitochondrial mutations on tumorigenesis, no signal study has related these mechanistically using the models available.

## OGlcNAcylation: A Rheostat Connecting Metabolic Dysregulation to Transcription

**Figure 3:**
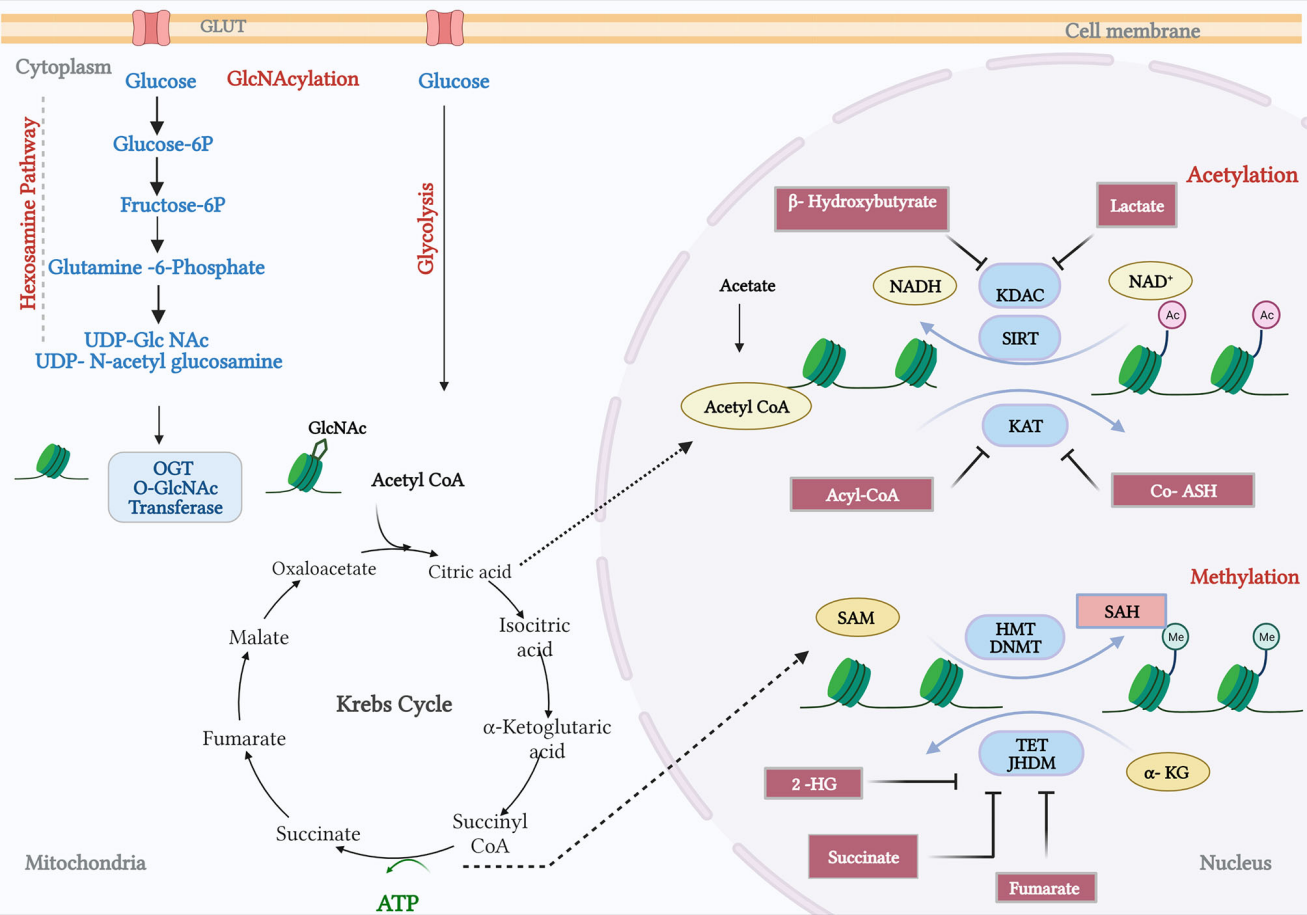
Crosstalk between metabolism and prostate cancer genomics. The hexosamine pathway catalyses the conversion of glucose and glutamine to provide a metabolite UDP-glucosamine (UDP-GlcNAc). The O-GlcNAc transferase (OGT) facilitates the N-acetylglucosamination (GlcNAcylation) or Glycation process. In addition, the Krebs cycle fuels metabolites private and citrate, which gets converted by acyl-COA synthetase and pyruvate dehydrogenase. Products from fatty acid oxidation, fatty Acyl CoA, inhibit the activity of lysine acetyltransferases (KAT). KAT reactions also release CoA-SH, which acts as an inhibitor. B-Hydroxybutyrate, a ketone from FA, and lactate, a by-product of glycolysis, have been shown to inhibit lysine deacetylase (KDAC). Amino acid metabolism and TCA cycle fuels in methionine and ATP, respectively, and synthesizes S-adenosylmethionine (SAM). Histone methyl transferases and DNA methyl transferases catalyse SAM to S-adenosylhomocysteine (SAH), which in turn can inhibit DNMTs and HMTs. TCA cycle intermediates, succinate, fumarate, α-ketoglutarate (α-KG), and 2-hydroxyglutarate (2-HG) acts as inhibitors of TET demethylases and Jumonji-C (JMJC) domain-containing histone demethylases (JHDMs). The figure has been adapted and modified from the review (101).

Histone acetylation and the OGlcNAcylation of chromatin are significant features of enhancers in the prostate cancer genome (101). Histone acetylation has been associated with increased lipid turnover under the influence of a number of the genomic drivers of prostate cancer that were discussed earlier, and particularly with PTEN-loss and Myc overexpression (28). OGlcNAcylation at enhancer sites occupied by c-Myc suggests that these metabolite-dependent modifications may sustain oncogenic activity in a feed-forward manner—transcriptionally driven metabolic dysregulation supporting oncogenic transcriptional activity (**Figure 3**). OGlcNAcylation, as a post-translational modification sustained by a metabolic adduct (UDP-GlcNAc), is an abundant feature of cancer cells and is, unlike other post-translational modifications, catalysed by a single enzyme, OGlcNAc transferase (OGT) (102, 103). UDP-GlcNAc is synthesised by the hexosamine biosynthesis pathway and utilised both by OGT and by enzymes in the endoplasmic reticulum for the N-linked glycosylation and proper folding of newly synthesised proteins. Consequently, whilst enzymes in the hexosamine biosynthesis pathway are AR dependent, and many are overexpressed in prostate cancer, there is not necessarily a direct relationship between their activity and the OGlcNAcylation status of OGT substrates (104). Indeed, recent studies indicate that hexosamine biosynthesis itself may restrain the emergence of castrate-resistant prostate cancer (105). OGT activity itself, however, can support cancer progression, and the challenge is in determining the nature of the substrates, and biological processes are the primary mediators of this crosstalk. There are a number of biologically compelling candidates including c-Myc, FOXM1, and HIF1a, all of which are known to be OGlcNAcylated and more active due to OGT function (106). There are also broader impacts of OGT activity; at the chromatin level, it is known to modify histones, and transcriptionally, it is known to regulate RNA polymerase II activation and processivity working in concert with cyclin-dependent kinases such as CDK7 and CDK9 (107). By implication, OGT activity reflects a nutrient-replete/”fed” state sufficient to permit glucose, glutamine, UTP, and acetyl-CoA to be used to provide adequate UDP-GlcNAc as a substrate for OGT (108). However, that alone is unlikely to provide an understanding of crosstalk; we need to establish more clearly how OGT selects protein substrates, and we also need to account for a second enzymatic activity ascribed to OGT, its proteolytic function. A recent study has indicated that in some cell types, OGT can sustain cell proliferation through a non-catalytic function that needs to be fully characterised (109). This in turn also needs to be put into a spatial context, since OGT can function as different isoforms in distinct organelles within the cell, mitochondria, and nuclei being principal examples. That final aspect is of course reminiscent of TCA cycle enzymes such as PDHA1 and ATP-citrate lyase as previously described in the context of acetylation.

Given that a limited number of metabolic pathways may be the mediators of feedback effects of oncogenes and tumour suppressors on chromatin/the epigenome, how do we use this knowledge to benefit patients? To achieve this, we arguably need to fill a knowledge gap in our ability to identify metabolites and understand how changes in metabolic activity occur spatially within tumours.

## Future Perspectives

As outlined in this article, there are significant examples of crosstalk between metabolic pathways and other cancer drivers; it remains challenging to prove that this crosstalk plays a causative role in prostate cancer progression. This is because changes in metabolic activity will inevitably impact on the redox state of the cell and the availability of metabolites for anabolic metabolism and sustain transcription and DNA replication. In addition, most metabolic processes are also contributors to the functions of untransformed cells in immune system and tumour micro-environment and are affected by the availability of nutrients and by crosstalk between ranges of cell types. In the big picture, how do we achieve cancer selectivity in modelling this interplay and in targeting this crosstalk?

First, an important factor to address is our lack of knowledge of the metabolome. As it stands, a metabolomics study can only identify maximally approximately 10% of the metabolite signals that are measurable using mass spectroscopy or other methods. This means that our understanding of the activity of mitochondria and metabolic pathways in cancer cells is constrained by our capacity to identify novel metabolites (“oncometabolites”) in a sensitive and unbiased manner. This missing information is by contrast increasingly an alien concept in the field of cancer genomics due to the capacity to sequence at high scale and decode genomic data. This has led, for example, to the discovery of highly cancer-specific non-coding transcripts and somatic DNA mutations; we have no equivalent signatures thus far in the cancer metabolome. Addressing this point is predominantly a technical challenge.

Second, clinical disease is by definition molecular heterogeneous. We know that this is true when we assess bulk sequencing data and look for high incidence mutations in multi-focal tumour samples from a single patient. However, recently, it has been possible to combine spatial information including pathology and genomic data using new platforms that permit RNA extraction, library preparation, and sequencing on a solid-phase surface/glass slide. This spatial dimension permits molecular information to be mapped onto distinct cell sub-populations and interpreted more readily in the context of associations between cell types at tumour–stromal interface and elsewhere. As a consequence, spatially resolved transcriptomics was declared to be the Method of the Year for 2020 by Nature Methods (110). Equivalent spatial resolution of those metabolic signals that we can attribute to known metabolites would provide great insights into cell–cell crosstalk in prostate cancers. This is important to test hypotheses, for example, around the compartmentalisation of metabolism in cancers, such as the idea of a lactate shuttle between cancer and stromal cells and compartmentalisation of glycolysis and oxidative phosphorylation between distinct cell types that communicate with each other in tumours (111). Significant progress is being made in developing single-cell spatial and *in situ* methods for the sensitive detection of well-known metabolites, and mass spectrometry imaging is showing promise in defining more complex and accurate metabolic classifiers of disease (112). However, there is a need for new devices/biomedical engineering to capture metabolic information in real time in an operating theatre, as the signals can be significantly affected by environmental factors. As matter of concern, the metabolomic signals are dynamic/unstable, and sequencing data (DNA) is stable, and technology needs to address those differences (113–117). With the vast increase in prostate cancer genomic data and other data types (clinical, pathological, imaging, metabolomics, and proteomics), there is a significant challenge in assimilating, refining, and deciphering biologically informative signals that reflect crosstalk. Machine-learning algorithms and artificial intelligence promise to alleviate this issue, since they are, in many cases, data-type agnostic. Their impact is exemplified in the sphere of medical imaging. Digital pathology, aided by artificial Intelligence (AI), has decoded large datasets to improve the reliability of diagnostic pathology and improve the prediction of treatment outcome and patient survival (118). Additionally, multiparametric MRI has significantly improved sampling of clinical significant prostate cancers at biopsy, and improvements in both the scanners and analytical approaches employed on the resultant data will further enhance and standardise this work across clinical centres (119). We can anticipate a future in which spatial genomic and metabolomic data are aligned to radiomic features and imaging to risk stratify patients and simultaneously inform treatment selection (119).

## Author Contributions

RS wrote the manuscript and made the figures. IM discussed the themes and provided advice during the drafting process. All authors contributed to the article and approved the submitted version.

## Conflict of Interest

The authors declare that the research was conducted in the absence of any commercial or financial relationships that could be construed as a potential conflict of interest.

## Publisher’s Note

All claims expressed in this article are solely those of the authors and do not necessarily represent those of their affiliated organizations, or those of the publisher, the editors and the reviewers. Any product that may be evaluated in this article, or claim that may be made by its manufacturer, is not guaranteed or endorsed by the publisher.
